# An Inguinal Stomach

**DOI:** 10.5334/jbsr.3244

**Published:** 2023-08-30

**Authors:** Cláudio Rodrigues, João Luís Pinheiro, Eugénia Cancela

**Affiliations:** 1 Centro Hospitalar Tondela-Viseu, PT

**Keywords:** stomach, inguinal hernia, computed tomography

## Abstract

**Teaching Point:** Although inguinal hernias are very common, stomach entrapment within an inguinal hernia is rare.

## Case Report

**Figure 1:**
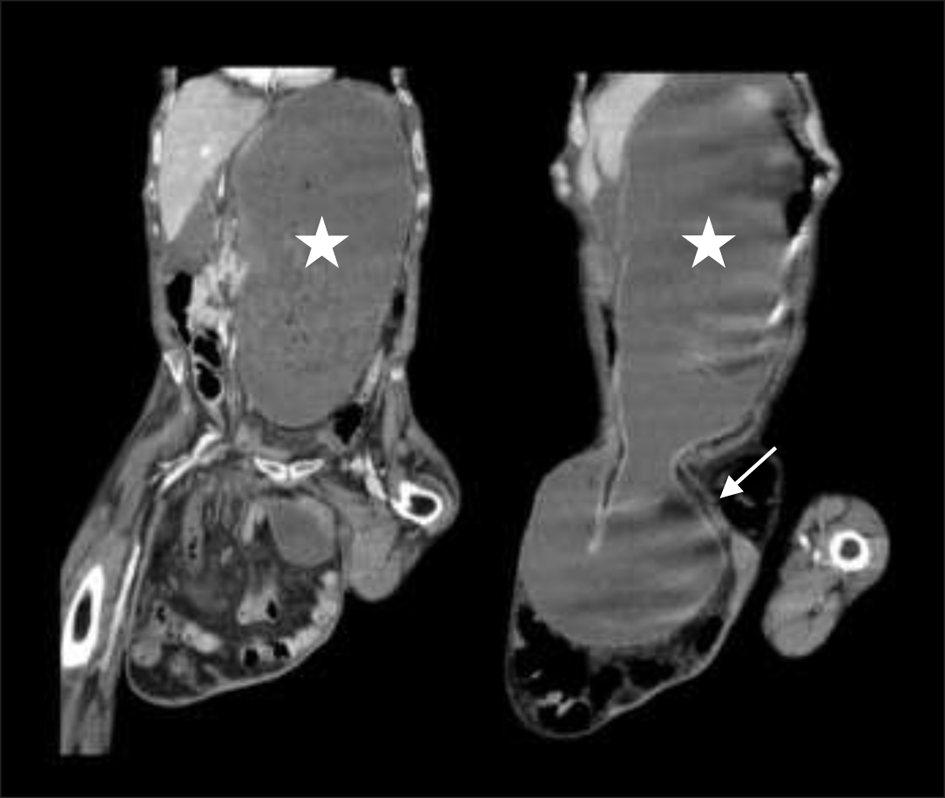


**Figure 2:**
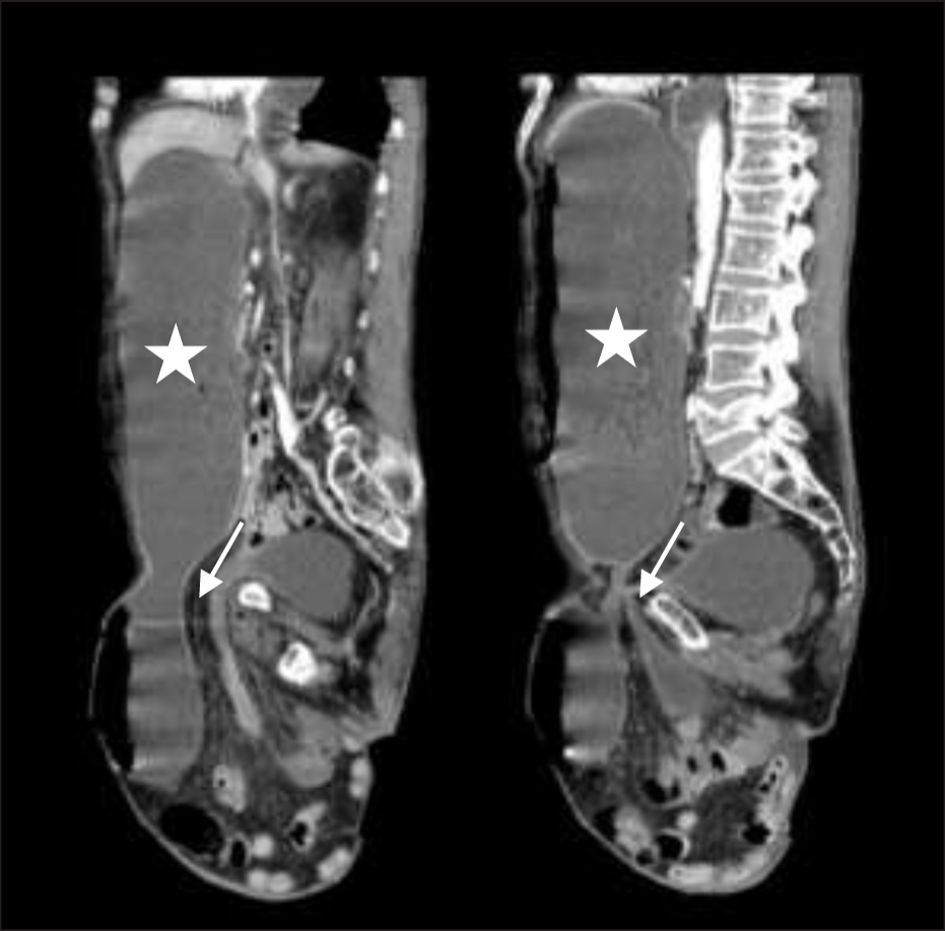


A 70-year-old man with history of multiple myeloma, hypothyroidism and type 2 diabetes mellitus was admitted in the emergency department for nausea and vomiting. Vital signs were stable. Clinical examination revealed no abdominal distension but a large irreducible right inguinal hernia, with mild pain. Laboratory findings showed neutrophilia with normal C-reactive protein level. An abdominal and pelvic computed tomography scan showed a large inguinal hernia (white arrow) containing a considerable part of the stomach (white star) (Figure 1, coronal reconstruction, and Figure 2, sagittal reconstruction). A significant stomach distension was observed but with no signs of ischemia. A small amount of free fluid was seen mainly in perihepatic topography, although pneumoperitoneum was not visible. Considering the patient comorbidities, medical treatment was undertaken with continuous gastric aspiration with nasogastric tube and intravenous hydration. However, on the second day after admission the patient presented with respiratory failure due to right aspiration pneumonia. Despite antibiotics and supportive measures, the patient passed away.

## Comments

Inguinal hernia with stomach content is uncommon, with few cases reported in the literature [1]. Computed tomography is generally needed to visualize the stomach within the hernia and exclude complications. Due to the rarity of this condition, there is no standard surgical procedure concerning the treatment. When feasible, a conservative management is deemed appropriate in patients with important comorbidities.
